# Biomarcadores de hipogonadismo masculino en la infancia y la adolescencia

**DOI:** 10.1515/almed-2019-0043

**Published:** 2020-04-04

**Authors:** Rodolfo A. Rey

**Affiliations:** Centro de Investigaciones Endocrinológicas “Dr. César Bergadá” (CEDIE), CONICET-FEI- División de Endocrinología, Hospital de Niños Ricardo Gutiérrez, Gallo, 1330, C1425EFD, Buenos Aires, Argentina

**Keywords:** ambigüedad genital, criptorquidia, disgenesia gonadal, hipogonadismo hipergonadotrófico, hipogonadismo hipogonadotrófico, micropene, testículo, trastornos del desarrollo sexual

## Abstract

El eje hipotálamo-hipófiso-testicular es activo en la vida fetal y durante los primeros meses de la vida posnatal: la hipófisis secreta hormona luteinizante (LH) y folículo-estimulante (FSH), mientras que el testículo produce testosterona y factor insulino-símil 3 (INSL3) en las células de Leydig y hormona anti-Mülleriana (AMH) e inhibina B en las células de Sertoli. En la infancia, los niveles séricos de gonadotrofinas, testosterona y factor INSL3 disminuyen a valores prácticamente indetectables, pero los de AMH e inhibina B permanecen altos. En la pubertad, se reactivan las gonadotrofinas y la producción de testosterona e INSL3, aumenta la inhibina y disminuye la AMH, como signo de maduración de la célula de Sertoli. Sobre la base del conocimiento de la fisiología del desarrollo del eje, es posible utilizar clínicamente estos biomarcadores para interpretar la fisiopatología y diagnosticar las diferentes formas de hipogonadismo que pueden presentarse en la infancia y la adolescencia.

## Introducción

Clásicamente se define al hipogonadismo masculino como el fallo testicular caracterizado por una deficiencia androgénica. Si bien esa definición es ampliamente aceptada en la endocrinología del adulto, tiene poca aplicación en el paciente pediátrico [1]. Para comprender las dificultades que pueden emerger de un uso inadecuado de la definición de hipogonadismo en niños y adolescentes, es necesario revisar la fisiología del desarrollo de eje hipotálamo-hipófiso-testicular (H-H-T).

## Fisiología del desarrollo del eje H-H-T

**Figura 1:**
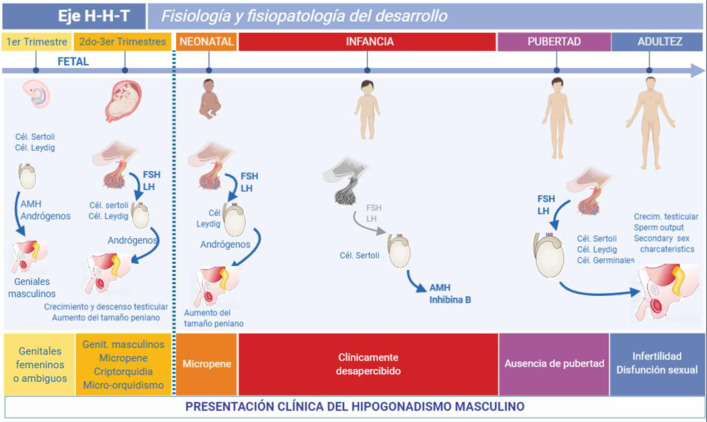
Ontogenia del eje hipotalámico-hipófisis-testicular (H-H-T) en el varón y su impacto en la presentación clínica del hipogonadismo. Las gónadas se diferencian en el primer trimestre de la vida fetal, independientemente de las gonadotrofinas hipofisarias. Los andrógenos testiculares y la hormona anti-Mülleriana (AMH) provocan la diferenciación masculina de los genitales; su ausencia conduce a la diferenciación femenina. El hipogonadismo en este período lleva al desarrollo de genitales ambiguos o femeninos en individuos XY. En los trimestres segundo y tercero, los andrógenos provocan el descenso testicular y el agrandamiento del pene. Los hipogonadismos primarios y secundarios dan como resultado micropene, microorquidismo y/o criptorquidia en un recién nacido que presenta genitales masculinos. En los primeros meses de la vida posnatal, la secreción de gonadotrofinas y andrógenos es activa; el hipogonadismo impide el agrandamiento del pene en los niños. Durante la infancia, las gonadotrofinas y la testosterona son normalmente bajas o incluso indetectables; el hipogonadismo establecido en este período no da lugar a signos clínicamente evidentes y sólo puede detectarse si se evalúan los niveles de AMH o inhibina B. Durante la pubertad, el eje H-H-T se reactiva y provoca el desarrollo típico de caracteres sexuales secundarios; el hipogonadismo puede provocar un desarrollo puberal ausente o incompleto, o más tarde infertilidad y disfunción sexual. Esta figura se modificó utilizando BioRender (https://biorender.com/) con permiso de Grinspon RP, Freire AV, Rey RA. Hypogonadism in Pediatric Health: Adult Medicine Concepts Fail. Trends Endocrinol Metab 2019;30(12):879–890. © 2019 Elsevier Ltd.

Los testículos se diferencian durante la sexta semana del desarrollo embrionario (8 semanas de amenorrea), antes de que el eje hipotálamo-hipofisario sea funcionalmente activo [2]. Los túbulos seminíferos se forman por la asociación de las células de Sertoli, que rodean a las células germinales; en el tejido intersticial, se diferencian las células de Leydig. Las células de Sertoli producen hormona anti-Mülleriana (AMH), responsable de la regresión de los conductos paramesonéfricos de Müller (esbozos del útero y las trompas de Falopio) en las semanas 8 y 9 de la vida intrauterina (Figura 1). En esa etapa, la producción de AMH es independiente de las gonadotrofinas hipofisarias pero, a partir de la segunda mitad de la gestación, responde a la hormona folículo-estimulante (FSH) [3]. Las células de Sertoli también secretan inhibina B, que responde a la FSH y es responsable de la retroalimentación negativa sobre la producción hipofisaria de FSH [4], [5]. Las células de Leydig producen andrógenos (Figura 1), que provocan el desarrollo de los conductos mesonéfricos de Wolff para formar los epidídimos, los conductos deferentes y las vesículas seminales, así como la virilización del seno urogenital y los esbozos de los genitales externos [6]. La síntesis de andrógenos depende esencialmente de la acción de la gonadotrofina coriónica humana (hCG) en el primer trimestre de la gestación y de la hormona luteinizante (LH) hipofisaria posteriormente. Las células de Leydig también secretan el factor insulino-símil 3 (INSL3) que es responsable, junto con los andrógenos, del descenso testicular hacia el escroto [7], [8].

Al nacimiento, las concentraciones de todas las hormonas del eje gonadal se encuentran bajas en sangre, pero aumentan progresivamente a partir de la primera semana de vida [9]. Las gonadotrofinas, la testosterona y el INSL3 permanecen en niveles similares a los del adulto hasta aproximadamente el sexto. mes de vida, en que disminuyen a niveles bajos o indetectables [10]. La AMH persiste en niveles elevados durante toda la infancia, reflejando el estado inmaduro de la célula de Sertoli [11], [12], mientras que la inhibina B disminuye parcialmente pero mantiene niveles claramente detectables [5]. Durante la infancia, el volumen testicular aumenta de manera imperceptible clínicamente, siendo la población de células de Sertoli la que mayor aporte hacen al tamaño gonadal [13]. Las células de Sertoli no maduran en los primeros 6 meses, a pesar de estar expuestas a altas concentraciones de andrógenos, dado que no expresan el receptor de andrógenos [14], [15], [16]. Luego del primer año comienzan a expresarlo, pero persisten inmaduras durante el resto de la infancia porque los niveles de testosterona son muy bajos [17]. Las células germinales sólo se dividen por mitosis, pero no entran en meiosis, por lo cual no hay producción espermática.

El inicio del desarrollo puberal se caracteriza por una reactivación del gonadotropo, que comienza a producir FSH y LH de manera cíclica. La FSH provoca una proliferación de las células de Sertoli aun inmaduras. Así, el volumen testicular comienza a aumentar progresivamente. La LH estimula la producción de testosterona por las células de Leydig; el aumento de la testosterona intratesticular induce la maduración de las células de Sertoli, que comienzan a producir menos AMH y más inhibina B [13]. Otra característica de las células de Sertoli maduras es que son capaces de desarrollar la barrera hemato-testicular y sostener funcionalmente a la espermatogénesis adulta [18]. La marcada proliferación de las células germinales es responsable del notable aumento del volumen testicular.

## Biomarcadores del eje H-H-T

### Hormonas hipofisarias: LH y FSH

Las gonadotrofinas LH y FSH tienen en común, junto con la tirotropina (TSH) hipofisaria y la hCG, la subunidad alfa y deben su especificidad a la subunidad beta. Ambas son secretadas por el gonadotropo hipofisario, en respuesta al estímulo que produce la hormona liberadora de gonadotrofinas (GnRH) producida a nivel del hipotálamo.

#### LH

La LH se une al receptor de LHCG (LHCGR), el cual responde tanto a la LH como a la hCG, y está presente en la membrana de las células de Leydig testiculares. La LH, de forma aguda, provoca un estímulo de la esteroidogénesis testicular, con el consecuente aumento en los niveles circulantes de testosterona. De forma crónica, la LH tiene un efecto trófico sobre las células de Leydig, aumento su número (hiperplasia) y su secreción de INSL3 [19], [20]. El descenso en los niveles de LH conlleva a una desdiferenciación de las células de Leydig a precursores mesenquimáticos y un descenso en los niveles de andrógenos e INSL3, tras el período de 3 a 6 meses de activación posnatal, clásicamente conocido como “mini-pubertad” [10], [13], [21]. En la pubertad, que en el varón puede iniciarse entre los 9 y los 14 años [22], la LH provoca nuevamente el desarrollo de células de Leydig y la producción de andrógenos e INSL3.

**Figura 2:**
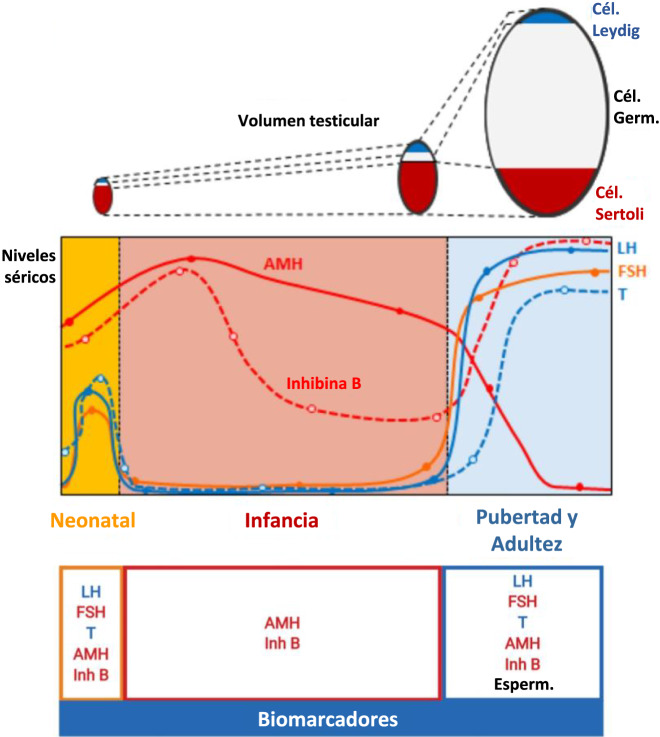
Ontogenia esquemática de la evolución del volumen testicular desde el nacimiento hasta la edad adulta. Los túbulos seminíferos (células de Sertoli + células germinales) son siempre el componente principal de los testículos. Desde el nacimiento y durante todo el período prepúber (es decir, hasta los 9–14 años, estadio 1 de Tanner), el volumen de túbulos seminíferos depende principalmente de las células Sertoli, mientras que el aumento significativo en el volumen testicular durante el desarrollo puberal (es decir, entre las etapas de Tanner 2 y 5) se debe principalmente a la proliferación de células germinales (espermatogénesis adulta). Esta figura se modificó utilizando BioRender (https://biorender.com/) con permiso de Grinspon RP, Freire AV, Rey RA. Hypogonadism in Pediatric Health: Adult Medicine Concepts Fail. Trends Endocrinol Metab 2019;30(12):879–890. © 2019 Elsevier Ltd.

Los niveles circulantes de LH son muy bajos en las primeras horas tras el nacimiento [23] y aumentan en la primera semana de vida [9] para permanecer en valores similares a los de la pubertad hasta los 3 a 6 meses de vida [10]. Luego, la LH sérica disminuye hasta valores generalmente no detectables por los métodos habitualmente utilizados en la clínica y permanece así hasta el inicio de la pubertad (Figura 2). Durante el desarrollo puberal, la LH comienza a ser secretada en pulsos cada aproximadamente 90 minutos, que son primero nocturnos para luego hacerse presentes durante todo el día [24]. Los niveles circulantes de LH aumentan progresivamente durante el desarrollo puberal, de acuerdo con los estadios de Tanner [12]. La evaluación de los niveles de LH, al igual que los del resto de las hormonas del eje gonadal, deben ser evaluados en función de los estadios de Tanner [25] y no de la edad, dada la amplia variabilidad interindividual que existe en cuanto a la edad de inicio y de fin de la pubertad en la población general [22].

#### FSH

La FSH se une a su receptor específico (FSHR), que está presente en la membrana de las células de Sertoli. La FSH tiene un efecto proliferativo sobre las células de Sertoli inmaduras (desde la vida fetal hasta el inicio de la pubertad), siendo así un determinante esencial del tamaño testicular en esas etapas de la vida. Además, la FSH estimula la secreción de AMH [26] e inhibina B [27]. Al igual que la LH, la FSH sérica está baja al nacer [23], luego aumenta durante la primera semana de vida [9]. En el varón la FSH suele estar algo más baja que la LH en la “mini-pubertad” [10] y en la pubertad verdadera [12]. Durante la infancia, la FSH también disminuye, pero menos que la LH (Figura 2). Eso hace que los niveles circulantes de FSH estén por encima de los de LH en esta etapa de la vida [12], [28].

### Hormonas testiculares

Los testículos tienen dos poblaciones celulares con capacidad endocrina: las células de Sertoli y las células de Leydig. La actividad endocrina de las células de Leydig es clínicamente informativa durante la activación posnatal o “mini-pubertad” y en la pubertad, mientras que la actividad de las células de Sertoli es la que debe evaluarse clínicamente en la infancia.

#### Testosterona

Los niveles circulantes de testosterona siguen las mismas variaciones que los de la LH (Figura 2): son bajos al nacer [23] y aumentan progresivamente durante el primer mes de vida [9]. Si bien es aconsejable medir los niveles de esteroides por espectrometría de masas, la mayoría de los laboratorios clínicos siguen usando actualmente inmunoensayos [29]; por ello, es importante tener en cuenta que en las primeras 2 a 3 semanas de vida, los inmunoensayos pueden detectar inespecíficamente otros esteroides, siendo fundamental hacer las determinaciones tras un proceso de extracción para evitar valores artificialmente elevados [9]. Después de los 3 a 6 meses de vida, la testosterona circulante suele ser no detectable en plasma, aumentando nuevamente recién en el estadio 2 o 3 de Tanner [12]. En la infancia, es factible medir la testosterona circulante tras un estímulo con hCG (2 a 3 inyecciones IM de 1500 a 2500 UI, con 48 horas de diferencia), para estimar la capacidad funcional de las células de Leydig [30].

#### INSL3

La secreción testicular de INSL3 muestra perfiles similares a los de testosterona [10], [13], [21]. Sin embargo, dado que refleja solamente el efecto trófico de larga duración de las gonadotrofinas sobre las células de Leydig, su medición no es tan útil luego del estímulo agudo con hCG [31].

#### AMH

La AMH es un marcador distintivo de la célula de Sertoli prepuberal (Figura 2). La AMH sérica disminuye transitoriamente al nacimiento, pero luego aumenta durante las primeras semanas de vida [9] para llegar a sus niveles máximos entre los 2 y 3 años de edad, siendo hasta 100 veces más elevada en el varón que en la mujer [11], [12], [32]. Ello es de importancia en la práctica ya que requiere una dilución de la muestra en varones para que la concentración de AMH entre en el rango de los inmunoensayos usados actualmente.

Aunque la producción basal de AMH es independiente de las gonadotrofinas [33], la FSH estimula la secreción testicular de AMH [26], [34], [35], [36]. Por su parte, el aumento de la concentración intratesticular de testosterona provoca una inhibición de la secreción de AMH [18], [37]. Es de interés notar que el aumento de los niveles circulantes de andrógenos provocados por el tratamiento farmacológico no logra niveles suficientes de testosterona intratesticular para provocar la caída de la AMH [38]. Asimismo, la AMH no disminuye en respuesta a la testosterona antes del año de vida dado que las células de Sertoli no expresan el receptor de andrógenos en ese período de la vida [14], [39].

#### Inhibina B

Las inhibinas son proteínas diméricas producidas por las gónadas [40], compuestas por una subunidad alfa común y una subunidad beta específica. La inhibina B, que contiene una subunidad beta B, es la única de relevancia fisiológica en el sexo masculino [41], [42]. La célula de Sertoli es la mayor fuente de inhibina B en el varón [4]; su producción es estimulada por la FSH [27], [35]. A su vez, la inhibina B es el principal inhibidor de la secreción hipofisaria de FSH. En caso de una secreción disminuida de inhibina B, o ausente como en la anorquia, los niveles de FSH se encuentran muy elevados. Sin embargo, durante la infancia, la FSH puede no estar elevada [28], lo que es un indicador del reposo hipotálamo-hipofisario (gonadotropo) en ese período de la vida.

Los niveles de inhibina B aumentan durante las primeras semanas de vida [9], llegando a niveles del adulto en el segundo año [43], [44]. Luego, en el resto de la infancia, los niveles son algo menores pero siempre claramente detectables (Figura 2) y superiores a los de las niñas. En la pubertad, la inhibina B aumenta nuevamente llegando a sus niveles máximos en el estadio 2 o 3 de Tanner [4], [44], [45]. A partir de ese momento, los niveles de inhibina B reflejan la actividad de las células de Sertoli y su interacción con las células germinales.

## Hipogonadismo masculino

La definición de hipogonadismo masculino, generada por la medicina del adulto [1], se refiere a la deficiente función testicular reflejada en insuficiencia androgénica acompañada o no de una deficiente producción espermática [46]. Si se aplicase esa definición en la infancia, analizando la fisiología del desarrollo previamente descripta, todos los niños deberían ser calificados como hipogonádicos ya que no producen testosterona ni espermatozoides. Sin embargo, las células de Sertoli son activas durante la infancia, provocando un aumento del tamaño testicular – si bien discreto (Figura 2) – y secretando AMH [47] e inhibina B [43], [44]. Como veremos, la valoración endocrina de las células de Sertoli es útil como indicador de función testicular en la población pediátrica. Una definición más integral de hipogonadismo masculino debe contemplar la función disminuida de la función testicular, comparada con la esperada para la edad, que puede involucrar a las células de Sertoli (AMH, inhibina B), de Leydig (testosterona, INSL3) y/o germinales [48].

Del concepto anterior, se desprende la idea de clasificar al hipogonadismo masculino no solamente según el nivel del eje H-H-T primariamente afectado sino también según el momento de la vida en que se establece y la población testicular primariamente dañada (Tablas 1 y 2).

**Tabla 1:** Hipogonadismo masculino de inicio fetal.

			Infancia					Pubertad-Adultez					
		Genitales	LH	FSH	T	AMH	Inh B	LH	FSH	T	AMH	Inh B	Esperm.
Hipogonadismo primario (Testicular)	Fallo gonadal generalizado												
	Disgenesia gonadal	Femeninos o ambiguos	N-A	N-A	B-ND	B-ND	B-ND	A	A	B-ND	B-ND	B-ND	Azoosp.
	Sme. Regresión testicular Torsión testicular	Micropene, Escroto vacío	N-A	N-A	B-ND	B-ND	B-ND	A	A	B-ND	B-ND	B-ND	Azoosp.
	Síndrome de Klinefelter, Varón XX	Masculinos	N	N	N	N	N	A	A	N-B	B-ND	B-ND	Azoosp.
	Fallo gonadal disociado												
	Células de Leydig												
	Hipoplasia/aplasia: Defectos esteroidogénicos	Femeninos o hipovirilizados	N-A	N	B-ND	N-A	N	A	A	B-ND	N-A	B-ND	Azoosp.
	Mutaciones de INSL3	Criptorquidia	N	N	N	N	N	N	N-A	N		N-B	Oligosp.
	Células de Sertoli												
	Mutaciones de FSH-R	Testículos pequeños	N	N	N	B	B	N	A	N	B	B	Oligosp.
	Mutaciones de AMH	PMDS	N	N	N	ND	N	N	N	N	ND	N	N
Hipogonadismo secundario (Central)	Fallo gonadal generalizado												
	Insuficiencia hipofisaria multihormonal	Micropene, Criptorquidia	B	B	B	B	B	B	B	B	B	B	Oligosp./azoosp.
	Hipogonadismo central aislado	Micropene, Criptorquidia	B	B	B	B	B	B	B	B	B	B	Oligosp./azoosp.
	Fallo gonadal disociado												
	Insuficiencia hipofisaria multihormonal	Testículos pequeños	N	B	N	B	B	N	B	N		B	
	Hipogonadismo central aislado: Mutaciones de *TAC3 o TACR3*	Micropene, Criptorquidia	B	N	B	N	N	B	N	B		B	Oligosp./azoosp.
	Mutaciones de *LHβ*	Micropene, Criptorquidia	B	N	B	N	N	B	A	B	A		Oligosp./azoosp.
	Mutaciones de *FSHβ*	Testículos pequeños	N	B	N			A	B	N			Oligosp./azoosp.
Hipogonadismo dual (Combinado)	Fallo gonadal generalizado												
	Síndrome de Prader-Willi Hipoplasia adrenal congénita ligada al X	Micropene, Criptorquidia	B-N	B-N	B-N	B	B	N	N	B	B	B	Oligosp./azoosp.

B, N, A, bajo, normal, alto en comparación con los niveles de referencia masculinos para la edad; ND, no detectable. Traducida con permiso de Rey RA, Grinspon RP, Gottlieb S, Pasqualini T, Knoblovits P, Aszpis S, Pacenza N, Stewart Usher J, Bergadá I, Campo SM. Male hypogonadism: an extended classification based on a developmental, endocrine physiology-based approach. Andrology. 2013;1(1):3–16. © 2012 American Society of Andrology and European Academy of Andrology.

**Tabla 2:** Hipogonadismo masculino de inicio postnatal.

		Infancia					Pubertad-Adultez					
		LH	FSH	T	AMH	Inh B	LH	FSH	T	AMH	Inh B	Espermatogénesis
Hipogonadismo primario (Testicular)	Fallo gonadal generalizado											
	Orquitis Torsión o traumatismo testicular	N	N	B-ND	B-ND	B-ND	A	A	B-ND	B-ND	B-ND	Oligosp./azoosp.
	Síndrome de down	N-A	N-A	N-B	N-B	N-B	A	A	B	B	B	Azoosp.
	Varicocele	N	N	N	N	N	N-A	N	N-B	N	N	Teratozoosp./asthenozoosp.
	Enfermedades crónicas: *Granulomatosas, amiloidosis, fibrosis quística, insuficiencia renal*						A	A	B-ND	B-ND	B-ND	Oligosp./azoosp.
	Hipogonadismo de comienzo tardío	No corresponde					N-A	N-A	B		B	
	Fallo gonadal disociado											
	Deleciones del cromosoma Y: *AZF* Mutaciones génicas: *CILD1*, *USP9Y*	N	N	N	N	N	N	A	N		B	Oligosp./azoosp.
	Quimioterapia Radioterapia abdómino-pelviana	N	N	N-B		N-B	N-A	A	N-B		B	Oligosp./azoosp.
	Tratamientos farmacológicos: *espironolactona, ketoconazol*	N	N	N-B			N-A	N-A	B			Oligosp.
Hipogonadismo secundario (Central)	Fallo gonadal generalizado											
	Lesiones hipofisarias y del SNC: *Tumores, histiocitosis, traumatismos, etc.*	B	B	B	B	B	B	B	B	B	B	Oligosp./azoosp.
	Hipogonadismo central funcional: *Enfermedades crónicas, abuso de drogas o alcohol, etc.*						B-N	B-N	B		B	Oligosp./azoosp.
Hipogonadismo dual (Combinado)	Fallo gonadal generalizado											
	Radioterapia craneana + Quimioterapia Intoxicación con plomo Consumo de marihuana Irradiación corporal total	B-N	B-N	B-N	B	B	B-N	B-N	B	B	B	Oligosp./azoosp.

B, N, A, bajo, normal, alto en comparación con los niveles de referencia masculinos para la edad; ND, no detectable. Traducida con permiso de Rey RA, Grinspon RP, Gottlieb S, Pasqualini T, Knoblovits P, Aszpis S, Pacenza N, Stewart Usher J, Bergadá I, Campo SM. Male hypogonadism: an extended classification based on a developmental, endocrine physiology-based approach. Andrology. 2013;1(1):3–16. © 2012 American Society of Andrology and European Academy of Andrology.

### Hipogonadismo primario (“hipergonadotrófico”), secundario (“hipogonadotrófico”) o dual

El hipogonadismo puede deberse a una afección primaria del hipotálamo o la hipófisis, o a un daño principal de las gónadas. En raros casos, tanto el eje hipotálamo-hipofisario como las gónadas están primariamente dañados al mismo tiempo, lo cual da origen a un hipogonadismo “dual” o combinado [48].

#### Hipogonadismo primario

El hipogonadismo primario (testicular o periférico), usualmente denominado “hipergonadotrófico” en la medicina del adulto [1], [46], se caracteriza por un daño primario de las gónadas. La insuficiente producción de inhibina B y testosterona impiden un normal retrocontrol negativo a nivel hipotálamo-hipofisario, lo cual lleva en algunas etapas de la vida a un aumento de las gonadotrofinas. Ya veremos que ello no ocurre siempre y que puede haber una disociación entre los niveles de LH y FSH. Ejemplos de hipogonadismo primario son el síndrome de Klinefelter, el síndrome de regresión testicular y la orquitis, entre otros.

#### Hipogonadismo secundario

El hipogonadismo secundario (hipotálamo-hipofisario o central), usualmente denominado “hipogonadotrófico” en la medicina del adulto [1], [46], se caracteriza por un daño primario del hipotálamo o de la hipófisis. La insuficiente producción de LH y FSH impiden un normal desarrollo de las células de Leydig y de los túbulos seminíferos (células de Sertoli y germinales). Dada la fisiología normal del desarrollo del eje H-H-T, este tipo de hipogonadismo puede ser muy difícil de diagnosticar en la infancia; también es posible encontrar en algunos casos una disociación entre los niveles de LH y FSH, como se describe más adelante. Ejemplos de hipogonadismo secundario son el síndrome de Kallmann, la insuficiencia hipofisaria multihormonal y la insuficiencia hipofisaria post-cirugía del sistema nervioso central, entre otros.

#### Hipogonadismo dual

Existen condiciones poco frecuentes en las que tanto el eje hipotálamo-hipofisario como las gónadas están primariamente dañadas; es decir, a diferencia de las formas primarias y secundarias, en el hipogonadismo dual, el fallo ocurre conjuntamente en ambos niveles, sin se una consecuencia de la otra. Ejemplos de hipogonadismo dual son el síndrome de Prader-Willi y el fallo gonadal en pacientes oncológicos tratados con quimioterapia y radioterapia craneana, entre otros.

### Fallo gonadal generalizado o disociado

#### Hipogonadismo generalizado

En estos casos, todas las poblaciones celulares del testículo están primariamente dañadas, llevando una deficiencia de todas las hormonas testiculares y de la producción de células germinales. Son ejemplos la disgenesia testicular y el síndrome de Kallmann (hipogonadismo hipogonadotrófico aislado con hiposmia).

#### Hipogonadismo disociado

A diferencia de los anteriores, las formas disociadas de hipogonadismo se caracterizan por un daño primario de una de las poblaciones celulares del testículo. Secundariamente, a menor o mayor plazo, las otras poblaciones pueden también verse afectadas en diferente grado. Ejemplo de ello son la hipolasia de células de Leydig por mutaciones del LHCG-R, la deficiencia de FSH por mutaciones del gen de la subunidad beta de FSH que afecta esencialmente a las células de Sertoli y el fallo gonadal post-quimioterapia que daña primariamente a las células germinales.

### Hipogonadismo de inicio fetal, infantil, puberal o en la adultez

Las manifestaciones clínicas del hipogonadismo dependen de la etapa de la vida en la que el fallo se establece. El hipogonadismo fetal establecido en el primer trimestre del desarrollo provoca un trastorno del desarrollo sexual (DSD, por sus siglas en inglés correspondientes a Disorders of Sex Development), que se manifiesta por la existencia de genitales ambiguos o femeninos al nacimiento [49]. La disgenesia gonadal es un ejemplo de hipogonadismo fetal generalizado, mientras que la hipoplasia de células de Leydig es una forma disociada. El hipogonadismo central no puede dar lugar a la ambigüedad genital, ya que la función de las células de Leydig en el primer trimestre de la vida fetal no depende de las gonadotrofinas hipofisarias sino de la hCG placentaria. El hipogonadismo establecido a partir del segundo trimestre de la vida fetal, sea testicular, central o dual, da lugar típicamente a micropene y criptorquidia en un varón sin ambigüedad genital [50], [51], [52]. Puesto que el eje H-H-T permanece activo durante los 3 a 6 primeros meses de vida posnatal [9], [10], este período representa una ventana de oportunidad para establecer el diagnóstico de hipogonadismo [50], [52].

El hipogonadismo establecido durante la infancia puede pasar desapercibido. Esto se debe, como hemos visto, a que la función H-H-T normalmente disminuye en el niño. La condición debe sospecharse y buscarse activamente (por ejemplo, mediante la medición de AMH o inhibina B en condiciones basales, o testosterona en respuesta a la estimulación con hCG); de lo contrario el diagnóstico se retrasa hasta la edad puberal [53].

En varones que llegan a la edad puberal, el hipogonadismo se caracteriza por la ausencia o la detención del desarrollo puberal normal [22], [25]. Debido a la insuficiencia androgénica, las características sexuales secundarias no se desarrollan: las proporciones del cuerpo son típicamente eunucoides (relación entre segmento superior/inferior del cuerpo <1, con una braza que supera en 6 cm a la talla), la voz sigue siendo aguda, la edad ósea se retrasa, y el volumen testicular no aumenta, lo que refleja la falta o la detención de la espermatogénesis.

El hipogonadismo que se establece en la adultez se caracteriza por una disminución de la libido, la impotencia y la oligo- o azoospermia [46]. En un porcentaje de hombres de mayor edad, se desarrolla una deficiencia androgénica leve, conocida como hipogonadismo de inicio tardío [54], que presenta síntomas similares a los del hipogonadismo en hombres jóvenes.

## Utilidad clínica de los biomarcadores del eje H-H-T en la infancia y la adolescencia

### Entre el nacimiento y los 3 a 6 meses de vida

En esta etapa de la vida todo el eje H-H-T está activo, por lo cual todas las hormonas pueden ser informativas.

#### Recién nacidos con genitales ambiguos o femeninos

En un recién nacido con genitales ambiguos, deben buscarse las causas de un DSD. En pacientes con cariotipo 46,XY la causa puede ser una disgenesia gonadal, o sea un hipogonadismo fetal primario generalizado de inicio en el primer trimestre. El hallazgo de niveles muy bajos de AMH, inhibina B, testosterona e INSL3 es esperable, con elevación de las gonadotrofinas [49], [52], [55]. En estos pacientes, los estudios por imágenes demuestran la presencia de útero y trompas por deficiencia de AMH. En cambio, cuando la ambigüedad genital se acompaña de una ausencia de restos müllerianos, puede tratarse de un hipogonadismo fetal primario disociado, con fallo exclusiva del sector leydigiano. Las causas pueden ser una hipoplasia de células de Leydig por mutación del LHCG-R o anomalías en alguna de las proteínas involucradas en la esteroidogénesis testicular [56]. Si bien la testosterona está baja y la LH alta, la AMH se encuentra en valores normales del rango masculino, lo que elimina el diagnóstico de disgenesia gonadal [57]. Si tanto la testosterona como la AMH están en valores masculinos normales, no existe hipogonadismo y la causa del DSD puede ser una insensibilidad a los andrógenos por mutaciones del receptor de andrógenos, una deficiente producción periférica de DHT por mutación del gen de la 5α-reductasa o bien una causa no endocrina [49], [55], [58]. En pacientes con anomalías del par de cromosomas sexuales (por ejemplo deleciones del brazo corto del cromosoma Y, 45,X/46,XY u otros mosaicismos con cromosoma Y presente), la causa del DSD es una disgenesia gonadal.

Una forma poco frecuente de DSD 46,XY es el síndrome de persistencia de los conductos de Müller (PMDS), que se presenta con criptorquidia y genitales masculinizados completamente. Las gonadotrofinas y testosterona están en el rango masculino normal, mientras que la AMH es muy baja o indetectable en la mutaciones del gen *AMH* y normal cuando la mutación está a nivel del receptor *AMHR2* [59]. En el primer caso estamos frente a un hipogonadismo fetal primario disociado, con afectación exclusiva de las células de Sertoli, mientras que en el segundo no hay hipogonadismo sino resistencia periférica a la AMH.

En recién nacidos con cariotipo 46,XX, la ambigüedad genital puede ser por un exceso de andrógenos de origen suprarrenal (por ejemplo, hiperplasia suprarrenal congénita) [60] o placentario (deficiencia de aromatasa) [61]; estos pacientes tienen ovarios y la AMH e inhibina B están en el rango femenino [57]. Pero la ambigüedad genital puede deberse al desarrollo de tejido testicular, ya sea en forma de ovotestes o testículos disgenéticos [62]. En los dos primeros casos, la AMH y la testosterona suelen estar en valores intermedios entre los masculinos y los femeninos, y las gonadotrofinas pueden estar elevadas, o incluso en el rango normal cuando existe suficiente tejido ovárico funcional. Existe también la posibilidad del nacimiento de un varón con cariotipo 46,XX y genitales masculinos normales. Estos casos, que se detectan por la discordancia con un eventual cariotipo hecho durante la gestación, presentan todas las hormonas del eje H-H-T en valores masculinos normales hasta la pubertad, como se describe más adelante.

#### Recién nacidos con micropene, criptorquidia Y/O micro-orquidismo

El micropene, la criptorquidia y/o el micro-orquidismo son signos de fallo en el eje H-H-T. Se trata de un hipogonadismo fetal establecido a partir del segundo trimestre, es decir después de que los genitales ya se diferenciaron en sentido masculino. La asociación de niveles bajos de LH, FSH, testosterona, INSL3, AMH e inhibina B son altamente sugestivos del diagnóstico de hipogonadismo fetal central (hipogonadotrófico) que afecta a todos los sectores del eje [35], [52], [63], [64], [65]. Pero el cuadro puede también deberse a un fallo testicular primario generalizado establecido a partir del segundo trimestre de la gestación: el síndrome de regresión testicular se caracteriza por niveles indetectables de las hormonas testiculares con elevación de las gonadotrofinas [52], [66].

### Entre los 6 meses Y la edad de la pubertad

En esta etapa de la vida, las gonadotrofinas, la testosterona y el INSL3 son muy poco informativos, siendo las hormonas producidas por las células de Sertoli las de mayor utilidad clínica.

Si la condición es congénita pero el diagnóstico se retrasó, es posible que no sea fácil confirmar la causa del problema. Los niveles bajos de AMH e inhibina B indican una deficiencia de células de Sertoli, pero puede ser difícil de establecer si el trastorno es primariamente testicular o secundario a un fallo hipotálamo-hipofisario. En efecto, las gonadotrofinas pueden normalizarse durante la infancia en pacientes con hipogonadismo primario (DSD disgenético o por disfunción leydigiana, o en el síndrome de regresión testicular o anorquia). Es decir que el hipogonadismo primario no es siempre “hipergonadotrófico” en niños durante la edad prepuberal [28]. Los valores de testosterona no se distinguen de los de un niño normal (son indetectables), a menos que se realice una prueba de estímulo con hCG, que busque definir si existen células de Leydig funcionales. Si la AMH y la inhibina B son indetectables, el diagnóstico de anorquia está confirmado.

En los niños sin antecedentes perinatales de micropene, la posibilidad de un hipogonadismo fetal está más alejada. La consulta puede ser motivada por criptorquidia, torsión o traumatismo testicular o tratamientos oncológicos que puedan afectar la función gonadal Nuevamente, las gonadotrofinas y la testosterona basales no son informativas: las gonadotrofinas no se elevan durante la infancia cuando la noxa afectó a las gónadas luego de los 6 meses de vida [28]. En pacientes sin gónadas palpables, un valor detectable de AMH [67] o inhibina B [68] asegura la existencia de gónadas en posición ectópica, al igual que un aumento de la testosterona luego de una prueba de estímulo con hCG [67]. Valores bajos de AMH [53], [67], [69], [70] o inhibina B [68], [69] indican una función testicular alterada. Valores dentro del rango normal pueden verse en niños con monorquia [71]. Los valores circulantes de INSL3 no son informativos en este grupo etario [72].

### En edad puberal

La ausencia de signos del desarrollo puberal indica una deficiencia androgénica. Si bien puede deberse a un hipogonadismo primario, es raro que un fallo testicular curse con una afectación del sector leydigiano tal que provoque una ausencia completa de secreción androgénica. En general en el hipogonadismo primario, se afecta más el sector tubular, lo cual se refleja en un tamaño testicular pequeño [73]. Ejemplo de ello son el síndrome de Klinefelter [74], [75], el varón XX [76] y los pacientes sometidos a quimioterapia [70], en los cuales hay inicialmente una afectación de la población de células germinales con niveles circulantes normales de hormonas testiculares y gonadotrofinas hasta el estadio 3 de Tanner del desarrollo puberal. Recién entonces el hipogonadismo primario se hace “hipergonadotrófico”.

Más frecuentemente, la ausencia de desarrollo puberal puede ser debida a una deficiencia hipotálamo-hipofisaria, o sea un hipogonadismo central congénito o adquirido, o simplemente a un retraso en la reactivación del eje o “retraso puberal simple” [22]. El diagnóstico diferencial suele no ser sencillo: una vez descartadas causas generales, tales como enfermedades sistémicas agudas o crónicas, la valoración de los niveles circulantes de las hormonas del eje H-H-T pueden ser o no informativas. Las gonadotrofinas en valores prepuberales no distinguen al hipogonadismo central del retraso puberal simple y es necesario hacer pruebas de estímulo con GnRH [77] o análogos [78]. El diagnóstico se facilita cuando hay otras deficiencias hipofisarias, que aumentan la probabilidad de un déficit gonadotrófico. La testosterona y el INSL3 también persisten en valores prepuberales, no siendo distintivos entre el hipogonadismo central y el retraso puberal simple [79]. En cambio, la AMH y la inhibina B podrían ser de utilidad, ya que están más bajas en pacientes con hipogonadismo central que en aquellos con retraso puberal simple [79], [80].

Al igual que el hipogonadismo primario, el hipogonadismo central puede afectar a todas las poblaciones celulares (“generalizado”) o bien afectar inicialmente a un solo sector del eje. Ejemplos de hipogonadismo central disociado son las mutaciones de TAC3 y su receptor TACR3 [81] o de la mutación de la subunidad beta de la LH [82], que cursan con LH baja pero FSH normal, y las mutaciones de la subunidad beta de la FSH [83], que presentan FSH disminuida y LH y andrógenos normales.

El hipogonadismo dual se caracteriza por tener una afectación concomitante del eje hipotálamo-hipofisario y de las gónadas. Pueden ser trastornos congénitos pero en los que el hipogonadismo es una manifestación tardía, como en el síndrome de Prader-Willi [84], [85] y en el asociado a hipoplasia adrenal congénita de comienzo tardío por mutación de DAX1 [86]. Alternativamente, puede tratarse de condiciones adquiridas, como es el caso de los pacientes sometidos a quimioterapia, que afecta primariamente al testículo, y radioterapia craneana, que afecta al hipotálamo. La característica de estos pacientes es que, a pesar de los niveles bajos de todas las hormonas gonadales, las gonadotrofinas no aumentan, o sea se comportan como un hipogonadismo eugonadotrófico.

## Conclusiones

El hipogonadismo puede tener origen fetal o postnatal, siendo sus manifestaciones clínicas variables según el momento de la vida en que se establece. Pueden afectarse todos los componentes funcionales del testículo o primariamente uno de ellos, llevando a manifestaciones clínicas y bioquímicas específicas. Finalmente, pueden dañarse primariamente las gónadas o el eje hipotálamo-hipofisario, y más raramente pueden estar ambos concomitantemente afectados. Las gonadotrofinas y los andrógenos son biomarcadores útiles en el recién nacido y en el varón de edad puberal, mientras que en la infancia son de mayor beneficio clínico las determinaciones de AMH e inhibina B.
